# Bu-Zhong-Yi-Qi pill alleviate the chemotherapy-related fatigue in 4 T1 murine breast cancer model

**DOI:** 10.1186/1472-6882-14-497

**Published:** 2014-12-15

**Authors:** Mingzi Ouyang, Yanyan Liu, Wei Tan, Ya Xiao, Keqiang Yu, Xiaomin Sun, Ying Huang, JingRu Cheng, Ren Luo, Xiaoshan Zhao

**Affiliations:** Department of Traditional Chinese Medicine, Nanfang Hospital, Southern Medical University, Guangdong, Guangzhou 510515 China; School of Traditional Chinese Medicine, Southern Medical University, Guangdong, Guangzhou 510515 China; Department of Traditional Chinese Medicine, Guangdong General Hospital, Guangdong Academy of Medical Sciences, Guangdong Geriatric Institute, Guangzhou, Guangdong 510080 China; Department of Personnel Section, Southern Medical University, Guangdong, Guangzhou 510515 China; The First Affiliated Hospital, Guangzhou University of Chinese Medicine, Guangzhou, 510405 China

## Abstract

**Background:**

Paclitaxel induced fatigue still remains underrecognized and undertreated, partly because of limited understanding of its pathophysiology and lack of effective treatments. This study is aim to evaluate the anti-fatigue effects and mechanism of Bu-Zhong-Yi-Qi pill in murine 4 T1 breast cancer mice were treated with paclitaxel.

**Methods:**

Breast cancer mice established with murine 4 T1 cells were randomly and repectively divided into five groups: negative control group (NC), tumor control group (TC), paclitaxel group (PTX), Bu-Zhong-Yi-Qi pill group (BZYQ) and Bu-Zhong-Yi-Qi pill plus paclitaxel group (BZYQ + PTX). The mice were administered for 21 days. During this period, the tumor volume, body weight and the weight-loaded swimming time were measured. After the last administration, all mice were sacrificed, weighted the tumor, measured immune cell cytokines and oxidative stress indicator. The remaining 10 mice in each group were observed for survival analysis.

**Results:**

Treatments with BZYQ + PTX and PTX significantly reduced the rates of tumor volume in comparison with TC starting on the 9th day and the 18th day respectively (P < 0.05-0.01), and presented decreased tumor weight compared to TC (P < 0.05-0.01). Compared with mice in TC group, the median survival time and the average survival time in BZYQ + PTX group, BZYQ group and PTX group were significantly prolonged (P < 0.05-0.01). The swimming time of the BZYQ + PTX group gradually increased, which is longer than the PTX group on Day 14 and Day 21 (P < 0.01). The level of TNF-α was lower in BZYQ + PTX group than PTX group (P < 0.01). The level of SOD activity in BZYQ + PTX group was lower than the NC group (P <0.01), but much higher than the PTX group (P < 0.01). The level of MDA of BZYQ + PTX group was higher than the NC group (P < 0.01), but significant lower than the PTX group (P < 0.01).

**Conclusions:**

BZYQ has the potential of alleviating paclitaxel chemotherapy-related fatigue in 4 T1 breast cancer mice by reducing the serum levels of TNF-α and modulating the level of MDA and the SOD activity.

## Background

Breast cancer is the most common cancer affecting women at all ages globally. With the development of detection and treatment, the number of women who survive from breast cancer has increased significantly in recent years. Five-year survival rates for localized breast cancer have climbed to 98%, resulting in an estimated 2.6 million North American women living in the aftermath of breast cancer [1]. As survival times increase, addressing the impact of breast cancer and its treatment on long-term outcomes have become increasingly important [2]. Fatigue is the most frequently reported side effect of any chemotherapy, including paclitaxel. It is reported that as many as 68% of breast cancer patients have fatigue during paclitaxel chemotherapy, and fatigue is a key reason for patient discontinuation of treatment [3]. After completion of treatment, as many as 70% of breast cancer patients report continued fatigue, which has been documented to persist for up to 10 years [4, 5]. Although various pharmacologic and nonpharmacological approaches have been studied, paclitaxel induced fatigue still remains underrecognized and undertreated, partly because of limited understanding of its pathophysiology and lack of effective treatments [6, 7]. Therefore, the development with more effective treatments would be instrumental in the ability to fight paclitaxel induced fatigue.

Chinese herbal formulae Bu-Zhong-Yi-Qi Decoction (“Bojungikki-tang” in South Korea or “Hochu-ekki-to” in Japanese) is composed of 10 species of medicinal plants (as shown in Table 1) [8], has been widely used in traditional medicine in China, Japan, Korea and so on. This herbal prescription has been identified as an effective medication to improve the quality of life and nutritional status [9]. Bu-Zhong-Yi-Qi Decoction is useful not only for the improvement of daily activity of chronic fatigue syndrome [10, 11], but also for the enhancement of anti-tumor effect [12–16]. Some clinical studies of Bu-Zhong-Yi-Qi Decoction have shown beneficial effects on cancer-related fatigue and quality of lives among cancer patients [17].

**Table 1:** **The components in Bu-Zhong-Yi-Qi pill**

Components	Ratio	Major chemical constituents
*Radix Astragali*	27.8%	Astragalus Polysaccharides, Astragaloside I-VIII,
*Radix Glycyrrhizae*	13.9%	Glycyrrhizic acid, glycyrrhetinic acid, Liquiritin
*Radix Bupleuri*	8.3%	Saikosaponin a, Saikosaponin d, Saikosaponin c, Saikochrome A
*Radix Angelicae Sinensis*	8.3%	Z-ligustilide, Ferulic Acid, Angelica Polysaccharides
*Radix Codonopsis*	8.3%	Lobetyolin, Lobetyolinin, tanshenoside I, Geniposide, syringin
*Rhizoma Atractylodis Macrocephalae*	8.3%	Butenolide I, Butenolide II, Biatractylolide, Atractylone
*Rhizoma Cimicifugae*	8.3%	27-Deoxyactein, Cimicifugic acids A-E, Ferulic acid, Isoferulic acid
*Pericarpium Citri Reticulatae*	8.3%	Hesperidin, Nobiletin, D-Limonene, β-myrcene
*Rhizoma Zingiberis Recens*	2.8%	Gingerol, Diarylheptanoids, α-pinene, β-phellandrene, Ginger flavonoids
*Fructus Jujubae*	5.6%	Zizyphus saponln)I-III, Jujuboside A, Jujuboside B, stepharine

Based on the anti-fatigue and antitumor effects, we supposed that Bu-Zhong-Yi-Qi pill could be an alternative therapy to chemotherapy-related fatigue, and as far as we know, there has not been studied for its effect on chemotherapy–related fatigue. In this study, we have focused on the weight-loaded swimming capability, the tumor growth, and the biochemical markers level of Bu-Zhong-Yi-Qi pill in 4 T1 murine breast cancer model treated with paclitaxel to evaluate whether Bu-Zhong-Yi-Qi pill has beneficial effects on chemotherapy–related fatigue.

## Methods

### Reagents

Paclitaxel (PTX) was purchased from Medisan Pharmaceutical Co., Ltd. (Harbin, China). Bu-Zhong-Yi-Qi pill (BZYQ) was acquired from Wanxi Pharmaceutical Co., Ltd. (Nanyang, China). It is constituted with ten herbs and their proportions were shown in Table 1. The fetal calf serum and RPMI-1640 medium were purchased from Jinuo Biological Pharmaceutical Co., Ltd. (Hangzhou, China). Reagent kits for the determination of TNF-α, IL-6 and IL-1β were purchased from Huamei Biotechnology Co. (Wuhan, China). Assay kits for the determination of SOD and MDA were acquired Beyotime Institute of Biotechnology (Haimen, China).

### 4 T1 cell culture

4 T1 murine mammary carcinoma cells which were acquired from Shanghai Institute of Biochemistry and Cell Biology of Chinese Academy of Sciences (Shanghai, China) were grown in tissue culture flasks at 37°C in humidified atmosphere of 5% CO_2_ and were maintained as monolayer cultures in RPMI 1640 containing 10% heat-inactivated fetal calf serum (FCS) and antibiotics (100 units/ml penicillin and 100 g/ml streptomycin).

### Animals

Female BALB/c mice (7 weeks old, 16 ~ 20 g) were obtained from Medical Laboratory Animal Center of Guangdong province (Approval No. SCXK (Yue) 2008–0002, Foshan, China). The mice were acclimatized for 1 week before use and housed at a room temperature of 23°C ± 1°C with a 12 h-light and 12 h-dark cycle (lights on from 6: 00 am to 6: 00 pm). Food and water were available ad libitum. Mice were treated in compliance with the current law and the Guiding Principles for the Care and Use of Laboratory Animals approved by Southern Medical University Animal Care and Use Committee (Approval No.2013058).

### Experimental design

Mice were taken weight-loaded swimming test before the experiment, and the mice which swam for long or short period were screened out. Then 30 mice were randomly chosen as the normal control group (NC). The rest mice were used for establishing tumor grafts model. A total of 1 × 10^5^ 4 T1 cells were injected in the right fourth mammary fat pad of BALB/c mice [18]. 120 mice whose tumor had a mean volume of 50 ~ 100 mm^3^ were chosen on Day 7, and were randomly divided into four groups, in each group, 30 mice randomly divided into three subgroups. The four groups that were named as the PTX group (PTX, i.p., every two days, 10 mg/kg, 10 ml/kg [18] and normal saline, p.o., once daily, 20 ml/kg) (PTX), the BZYQ group (BZYQ, p.o., once daily, 1.5 g/kg, 20 ml/kg, 10x patient’s clinical dosage (same as clinical equivalent dose) and normal saline, i.p., every two days, 10 ml/kg.) (BZYQ), the BZYQ (p.o., once daily, 1.5 g/kg, 20 ml/kg) plus PTX (i.p., every two days, 10 mg/kg, 10 ml/kg) group (BZYQ + PTX) and the tumor control group (TC). The TC and the NC groups received normal saline in volumes equivalent to those used for injection and intragastric administration of the drugs. The duration of treatment lasts 21 days. Tumors sizes of subgroup 1 of each group were measured every three days using calipers and tumor volume was calculated with the use of the following formula: tumor volume (mm^3^) = L × W^2^ × 0.52, where L is the longest diameter, W is the perpendicular short diameter. And body weight was weighed every 7 days. At the end of the experiments, mice were fasted for 8 hours, and then the mice were anesthetized with pentobarbital sodium and sacrificed. Tumors were excised from the mice and weighed. The subgroup 2 were taken out from each group for weight-loaded swimming test, and the subgroup 3 in each group were observed for survival analysis.

### Weight-loaded swimming test

The subgroup 2 were taken out from each group for weight-loaded swimming test every 7 days. The procedure used in this experiment was similar to that described by Porsolt RD, et al. [19]. Briefly, 60 min after the last therapy, the mice were placed individually in a swimming pool (30 cm high, 25 cm in diameter) in which the mice could only support themselves by touching the bottom with their feet (at 25°C ± 1°C). A tin wire (7% of body weight) was loaded on the tail root of each mouse. The swimming period was regarded as the time spent by the mouse floating in the water with struggling and making necessary movements until exhausting its strength. The mice were assessed to be exhausted when they failed to rise to the surface of water to breathe within a 10 s period. At the end of the session, the mice were taken out from the water, dried with a paper towel, and placed back in their home cages. Water in the container was drained after each session.

### Determination of serum IL-1β, TNF-α and IL-6

At the end of the experiments, mice were fasted for 8 hours, and then the mice were anesthetized with pentobarbital sodium and sacrificed. The blood samples of the mice were collected in tubes without anticoagulant by removing the left eyeball. Serum was prepared by centrifugation at 1000 × g, 4°C for 15 min. The blood serum was tested to determine the concentration of IL-1β, TNF-α and IL-6. The concentration of IL-1β, TNF-α and IL-6 were tested using the following the recommended procedures which were provided by the kits.

### Determination of muscle superoxide dismutase(SOD) activity and malondialdehyde (MDA)

After the blood was collected, the gastrocnemius muscle of the mice was immediately dissected, homogenized with 20 mm Hepes buffer and centrifuged at 1400 × g, 4°C for 5 min. The resulting supernatant was removed and stored at -80°C for the following analysis. The levels of SOD activities and MDA were measured with a kit following the manufacturer’s protocol.

### Statistical analysis

All data were expressed as the mean ± standard deviation (SD) in the tables and indicated by vertical bars in the figures. Differences between groups were determined by ANOVA and Student’s t-test. For the survival time of animals, Kaplan-Meier curves were established for each group, and the survivals were compared by the log-rank test. Probability value P less than 0.05 was considered significant.

## Result

### Effects of body weight

**Figure 1:**
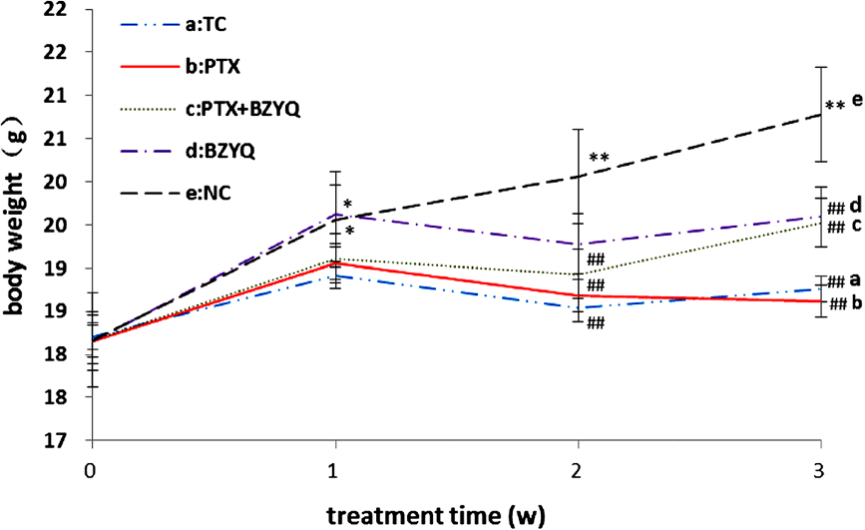
**Effects on body weight of mice.** Compared with the baseline. * P < 0.05, **P < 0.01. Compared with the NC, ^##^P < 0.01. n = 10.

Changes in mice body weights were recorded both before and after the treatment. The results are shown in Figure 1. The body weight was measured at the beginning of the experiment (week 0) and 3 weeks after treatment regular intervals. It was found that the body weight of the NC and BZYQ groups was obviously increasing compared with the baseline in week 1(P < 0.05) and the other three groups did not show any obvious changes (P > 0.05). The weight showed lower for TC, BZYQ + PTX and PTX groups than the NC group after 2 weeks (P < 0.01), and the body weight of the other four groups were all declining compared with the NC group after 3 weeks(P < 0.01), but there were no great difference among the four groups.

### Effects on tumor’s weight and volume

**Figure 2:**
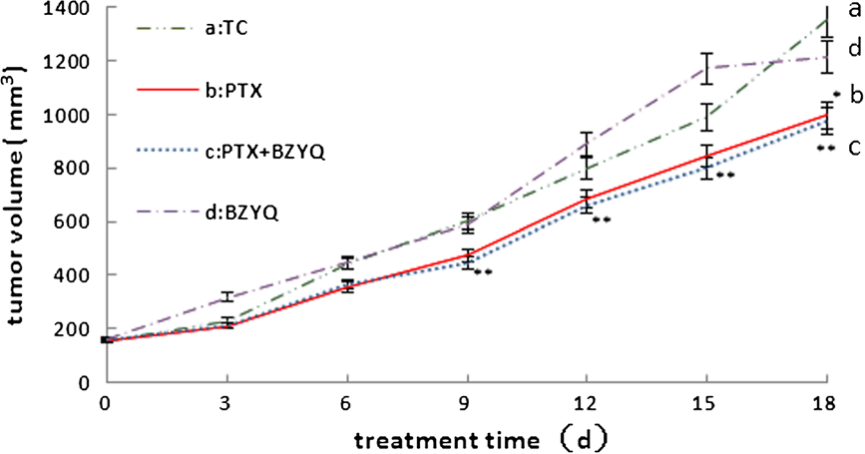
**Tumor’s volume.** Compared with TC, *P < 0.05, **P < 0.01. n = 10.

**Figure 3:**
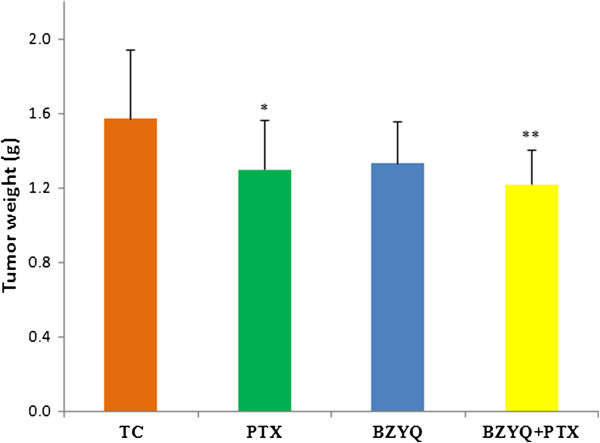
**Tumor’s weight.** Compared with TC, * P < 0.05, **P < 0.01. n = 10.

We examined the antitumor activity of BZYQ on an implant model of 4 T1 breast cancer separately and in combination with PTX, measured tumor volume at regular intervals and weighted the tumor after the mice were sacrificed after treatment lasts 21 days. As shown in Figures 2 and 3, treatments with BZYQ + PTX and PTX significantly reduced the rates of tumor volume in comparison with TC starting in Day 9 (P < 0.01) and Day 18 respectively (P < 0.05), and presented decreased tumor weight compared to TC (BZYQ + PTX, P < 0.01. PTX, P < 0.05), but the difference between the two groups was not significant (P > 0.05). Compared to TC, the rates of tumor growth did not reduce obviously in BZYQ with separate treatment (P > 0.05).

### Effects on the survival time of tumor-bearing mice

**Figure 4:**
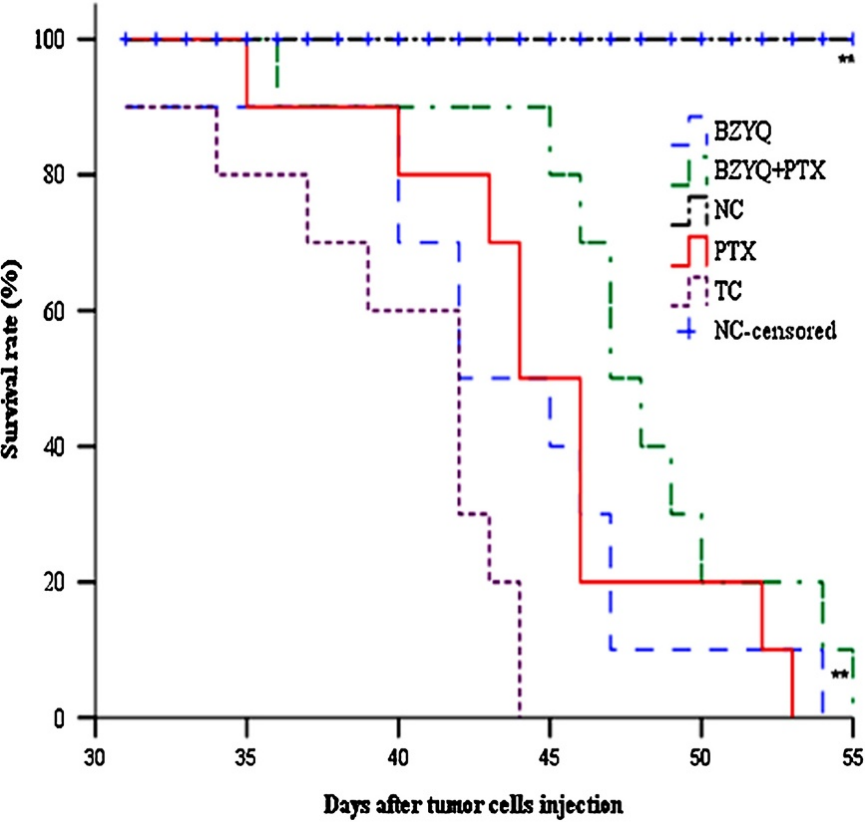
**Effect on the survival time of tumor-bearing mice.** Compared with TC, **P < 0.01. n = 10.

As shown in Figure 4. Compared with mice in TC group, the median survival time and the average survival time in BZYQ + PTX group, BZYQ group and PTX group were significantly prolonged (P < 0.05-0.01). Compared with mice in PTX group, the median survival time and the average survival time in BZYQ + PTX group were not significantly prolonged (P > 0.05).

### Effects on the weight-loaded swimming test

**Figure 5:**
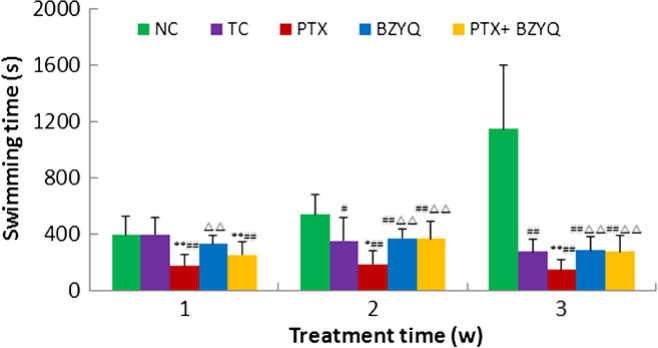
**Weight-loaded swimming time of all the groups.** Compared with the TC, *P < 0.05, **P < 0.01. Compared with the NC, ^#^P < 0.05, ^##^P < 0.01. Compared with the PTX, ^Δ^P < 0.05, ^ΔΔ^P < 0.01. n = 10.

The anti-fatigue activity of the BZYQ was measured as the swimming endurance capacity of 4 T1 breast cancer mice. Change of swimming time during the experimental period was shown in Figure 5. The swimming time of all groups had no significant difference before the intervention (P > 0.05). The NC group presented gradually increasing swimming time, while the TC group showed a declining swimming time, the swimming time of the PTX group and the BZYQ + PTX group were significantly decreasing after the treatment compared with the NC, TC and BZYQ groups (P < 0.05 ~ 0.01) in week 1. The swimming time of the BZYQ + PTX group gradually increased and was longer than the PTX group in week 2 and week 3 (P < 0.01), consistent with the TC and BZYQ group.

### Effects on serum TNF-α, IL-6 and IL-1β

As shown in Table 2, mice bearing breast cancer tumors exhibited higher levels of TNF-α, IL-6 and IL-1β of than the NC group (P < 0.05-0.01). The BZYQ + PTX group showed a lower level of TNF-α than the PTX group (P < 0.01), but the levels of IL-6 and IL-1β between the two groups had no significant difference (P >0.05).

**Table 2:** **Serum levels of TNF-α, IL-6 and IL-1β in mice (n = 10)**

Groups	TNF-α (pg/mL)	IL-6 (pg/mL)	IL-lβ (pg/mL)
NC	120.22 ± 6.41	21.77 ± 1.01	108.03 ± 6.24
TC	127.32 ± 5.54^#^**	23.27 ± 1.60^#^**	114.94 ± 7.05^#^*
PTX	142.30 ± 8.79^##^	25.43 ± 1.49^##^	122.21 ± 7.45^##^
BZYQ	129.92 ± 9.42^#^**	24.07 ± 1.24^##^*	115.55 ± 5.94^#^*
BZYQ + PTX	132.33 ± 11.09^##^*	26.62 ± 2.45^##^	121.26 ± 11.04^##^

Compared with the NC, ^#^P < 0.05,^##^P < 0.01. Compared with the PTX, *P < 0.05,**P < 0.01.

### Effects on muscle SOD activity and MDA

To gain better insight into the underlying mechanisms of BZYQ’s effect, we detected changes of SOD activity and MDA content in mice muscle. As shown in Table 3, the PTX group showed a decreased SOD activity compared with the other four groups (P < 0.01), the level of SOD activity of BZYQ + PTX group was lower than the NC group (P <0.01), but much higher than the PTX group (P < 0.01). The PTX group showed an increased MDA level compared with the other four groups (P < 0.01), the level of MDA of BZYQ + PTX group was higher than the NC group (P < 0.01), but much lower than the PTX group (P <0.01).

**Table 3:** **Muscle levels of MDA and SOD activity in mice (n = 10)**

Groups	MDA (nmol/mgPr)	SOD (U/mgPr)
NC	5.16 ± 0.80	55.08 ± 4.33
TC	6.18 ± 0.73^##^**	50.46 ± 3.16^#^**
PTX	8.30 ± 1.30^##^	33.91 ± 3.12^##^
BZYQ	6.33 ± 0.65^##^**	50. 43 ± 2. 30^##^**
BZYQ + PTX	6.99 ± 0.81^##^*	47.26 ± 3. 80^##^**

Compared with the NC, ^#^P < 0.05, ^##^P < 0.01. Compared with the PTX, *P < 0.05,**P < 0.01.

## Discussion

Fatigue is one of the most common side effects of paclitaxel chemotherapy which not only compromises patients’ quality of life, but also diminishes physical activity, resulting in limited treatment and increased morbidity. However, there were very few pharmacological drugs or therapies available for the treatment of chemotherapy-related fatigue. In this study, we used BALB/c mice that were treated with PTX in a tumor-bearing state to mimic a chemotherapy-related fatigue situation, the weight-loaded swimming time of the mice was used for the evaluation of fatigue related to chemotherapy. And we used the model to evaluate the effect of Bu-Zhong-Yi-Qi pill on the fatigue caused by paclitaxel. In our model, the weight-loaded swimming time was significantly reduced in response to administration of PTX starting in the first week after treatment and persisting for as long as 3 weeks consistent with the prior studies [20]. The swimming time of the BZYQ + PTX group gradually increased from week 2 and longer than the PTX group, consistent with the TC and BZYQ group. It is indicated that BZYQ enhanced the swimming capacity of PTX treated tumor-bearing mice confirming the anti-fatigue property of BZYQ.

It has been speculated that the fatigue that occurs in cancer patients during treatment with paclitaxel chemotherapy may be caused by the increase in production of inflammatory cytokines, and several studies have reported that paclitaxel is capable of inducing the expression of TNF-α, IL-1β, IL-6, IL-8, ICAM-1, NO synthetase, metaloproteinases and MCP-1 and causes the phosphorylation of MAP kinases [21]. Paclitaxel up-regulates IL-8 and IL-6 expression in human lung [22] and ovarian carcinoma cell lines [23]. It increases expression of IL-1β in human monocytes and in breast cancer cell lines, MCF-7 and ZR-75-1 [24]. It also has endotoxin-like effects on human macrophages inducing the release of IL-1β and TNF-α [25]. In these cytokines, the TNF-α, IL-1β and IL-6 play important roles in the development of fatigue [26–29]. Reports demonstrated that the Bu-Zhong-Yi-Qi pill could improve systemic inflammation and nutritional status by reducing the production of TNF-α and IL-6 [30–33]. In the study, we observed that PTX induced increased levels of TNF-α, IL-1β and IL-6, and the BZYQ could reduce the serum level of TNF-α but cannot decrease the levels of IL-1β and IL-6 up-regulated by PTX. These observations suggest that the level of TNF-α may be correlated with the anti-fatigue effect of BZYQ because of the possibility that elevated TNF-α level can lead to increased oxidants and muscle weakness in skeletal muscle according to mediate the majority of signaling through TNFR1 [34–36].

Growing evidence indicates that exposure of elevated oxidants is known to cause muscle weakness and accelerate the development of fatigue [37–39]. Except for being produced by tumors themselves [40], the oxidative stress state is always directly or indirectly produced by numerous chemotherapeutic agents. Some chemotherapeutic agents that include a quinone moiety in their chemical structure can directly produce a state of oxidative stress by interacting with molecular oxygen and undergoing redox cycling, leading to the generation of reactive oxygen species (ROS) [41]. Other chemotherapeutic agents can indirectly produce a state of oxidative stress by decreasing antioxidant levels, crippling the cell’s defenses against elevated oxidants [42]. In the present study, we found that the PTX group demonstrated a significantly decreasing muscle SOD activity and higher MDA level compared with the NC and TC groups, while the BZYQ + PTX group up-regulated the SOD activity and decreased the levels of MDA to that of the PTX group. On the other hand, the TC and BZYQ groups also demonstrated a significantly decreasing muscle SOD activity and higher MDA level compared with the NC group, but there was no significant difference between the two groups. Lipid peroxidation, which is a normal phenomenon that occurs continuously at low level is the most common consequence of oxidative stress. MDA is the last product of lipid peroxidation and is toxic to cells and cell membranes [43]. The natural cellular antioxidant enzymes SOD is accepted one of the most important physiological antioxidants against free radicals which prevent subsequent lipid peroxidation by speeding their dismutation [44]. As shown in a prior study that BZYQ can partly protect the gastric mucosa from ethanol-induced acute gastric injury by increasing the antioxidant status [45]. Radix Astragali, Radix Bupleuri and Glycyrrhizae, the major components of BZYQ, have been reported their anti-oxidative properties. Astragaloside IV (AS-IV), the active component of Radix Astragali, acted as an antioxidant inhibiting ROS generation and the reducing LPO content in culture-activated HSCs [46]. Saikosaponins are isolated as active ingredients of Radix Bupleuri, showed potent anti-inflammatory activities [47], anti-ulcerative [48], platelet activation inhibitory [49], corticosterone secretory [50], hepatoprotective [51], and nephroprotective [52] activities through their potent free radical scavenger effects, the antioxidant effects. Dehydroglyasperin D (DGD) is isolated from Glycyrrhizae also possessed the most potent antioxidant activity [53]. The present results suggested that BZYQ might effectively inhibited lipid peroxidation in muscle tissues, and acted directly or interacted with endogenous antioxidants for synergistic effects to defend against fatigue caused by PTX treated. But this effect looked like not obvious in the mice which did not treated with PTX, the difference of the mechanism of the oxidative stress caused by tumors and chemotherapeutic agents need to be further study. And we need to find out which element of BZYQ act the most important anti-chemotherapy-related-fatigue effect in the future studies.

Importantly, the BZYQ + PTX and PTX groups resulted in smaller tumor volume and less tumor weight compared with the TC group, moreover the mice of the BZYQ + PTX group had longer median survival times than the TC group; though there was no significant difference, the BZYQ + PTX group presented a tendency of less tumor weight, longer median survival time and heavier body weight than the PTX group. On the other hand, the tumor weight of the BZYQ and the TC groups had no significant difference, but the BZYQ group had a tendency of decreased tumor weight, longer median survival times and heavier body weight than the TC group. Tumor is a kind of wasting disease [54], and gastrointestinal dysfunctional is one of the serious side effects of PTX [55], in this study, we can assume that BZYQ has the potential effects on antitumor, improving quality of life and increasing body weight, which is consistent with the prior studies [9–17], we need enlarge the sample size to confirm the hypothesis in the next step.

## Conclusions

In conclusion, the present study has demonstrated the potential of BZYQ in alleviating paclitaxel chemotherapy-related fatigue by reducing the serum level of TNF-α and modulating the levels of MDA and SOD activity in the 4 T1 murine breast cancer model.

## Abbreviations

BZYQ: Bu-Zhong-Yi-Qi pill
NC: Negative control
TC: Tumor control
PTX: Paclitaxel group
BZYQ + PTX: Bu-Zhong-Yi-Qi pill plus paclitaxel
TNF-α: Tumor necrosis factor-α
IL-6: Interleukin-6
IL-1β: Interleukin-1β
SOD: Superoxide dismutase
MDA: Malondialdehyde.

## References

[1] Jemal A, Siegel R, Ward E, Hao Y, Xu J, Thun MJ (2009). Cancer statistics, 2009. CA Cancer J Clin.

[2] Ganz PA (2009). Survivorship: adult cancer survivors. Prim Care.

[3] Wang SH, He GP, Jiang PL, Tang LL, Feng XM, Zeng C, Wang GF (2013). Relationship between cancer-related fatigue and personality in patients with breast cancer after chemotherapy. Psychooncology.

[4] Ahles TA, Saykin AJ, McDonald BC, Furstenberg CT, Cole BF, Hanscom BS, Mulrooney TJ, Schwartz GN, Kaufman PA (2007). Cognitive function in breast cancer patients prior to adjuvant treatment. Breast Cancer Res.

[5] Alikunju S, Pillarisetti S (2006). Selected players in the inflammation cascade and drugs that target these inflammation genes against metastasis. Anticancer Agents Med Chem.

[6] Sood A, Moynihan TJ (2005). Cancer-related fatigue:an update. CurrOncol Rep.

[7] Dimeo FC (2001). Effects of exercise on cancer-related fatigue. Cancer.

[8] China PCOT (2005). Pharmacopoeia of the People's Republic of China, Part I.

[9] Tatsumi K, Shinozuka N, Nakayama K, Sekiya N, Kuriyama T, Fukuchi Y (2009). Hochuekkito improves systemic inflammation and nutritional status in elderly patients with chronic obstructive pulmonary disease. J Am Geriatr Soc.

[10] Chen R, Moriya J, Luo X, Yamakawa J, Takahashi T, Sasaki K, Yoshizaki F (2009). Hochu-ekki-to combined with interferon-gamma moderately enhances daily activity of chronic fatigue syndrome mice by increasing NK cell activity, but not neuroprotection. Immunopharmacol Immunotoxicol.

[11] Chen R, Moriya J, Yamakawa J, Takahashi T, Li Q, Morimoto S, Iwai K, Sumino H, Yamaguchi N, Kanda T (2008). Brain atrophy in a murine model of chronic fatigue syndrome and beneficial effect of Hochu-ekki-to (TJ-41). Neurochem Res.

[12] Utsuyama M, Seidlar H, Kitagawa M, Hirokawa K (2001). Immunological restoration and anti-tumor effect by Japanese herbal medicine in aged mice. Mech Ageing Dev.

[13] Li T, Tamada K, Abe K, Tada H, Onoe Y, Tatsugami K, Harada M, Kubo C, Nomoto K (1999). The restoration of the antitumor T cell response from stress-induced suppression using a traditional Chinese herbal medicine Hochu-ekki-to (TJ-41:Bu-Zhong-Yi-Qi-Tang). Immunopharmacology.

[14] Harada M, Seta K, Ito O, Tamada K, Li T, Terao H, Takenoyama M, Kimura G, Nomoto K (1995). Concomitant immunity against tumor development is enhanced by the oral administration of a kampo medicine, Hochu-ekki-to (TJ-41: Bu-Zhong-Yi-Qi-Tang). Immunopharmacol Immunotoxicol.

[15] Ito H, Shimura K (1985). Studies on the antitumor activity of traditional Chinese medicines. Gan To Kagaku Ryoho.

[16] Ito H, Shimura K (1985). Studies on the antitumor activity of traditional Chinese medicines. (II). The antitumor mechanism of traditional Chinese medicines. Gan To Kagaku Ryoho.

[17] Jeong JS, Ryu BH, Kim JS, Park JW, Choi WC, Yoon SW (2010). Bojungikki-tang for cancer-related fatigue: a pilot randomized clinical trial. Integr Cancer Ther.

[18] Dey JH, Bianchi F, Voshol J, Bonenfant D, Oakeley EJ, Hynes NE (2010). Targeting fibroblast growth factor receptors blocks PI3K/AKT signaling, induces apoptosis, and impairs mammary tumor outgrowth and metastasis. Cancer Res.

[19] Porsolt RD, Bertin A, Jalfre M (1977). Behavioral despair in mice: a primary screening test for antidepressants. Arch Int Pharmacodyn Ther.

[20] Ray MA, Trammell RA, Verhulst S, Ran S, Toth LA (2011). Development of a mouse model for assessing fatigue during chemotherapy. Comp Med.

[21] Penson RT, Kronish K, Duan Z, Feller AJ, Stark P, Cook SE, Duska LR, Fuller AF, Goodman AK, Nikrui N, MacNeill KM, Matulonis UA, Preffer FI, Seiden MV (2000). Cytokines IL-1beta, IL-2, IL-6, IL-8, MCP-1, GM-CSF and TNFalpha in patients with epithelial ovarian cancer and their relationship to treatment with paclitaxel. Int J Gynecol Cancer.

[22] Collins TS, Lee LF, Ting JP (2000). Paclitaxel up-regulates interleukin-8 synthesis in human lung carcinoma through an NF-kappaB-and AP-1 dependent mechanism. Cancer Immunol Immunother.

[23] Lee LF, Li G, Templeton DJ, Ting JP (1998). Paclitaxel(Taxol)-induced gene expression and cell death are both mediated by the activation of c-Jun NH2-terminal kinase(JNK/SAPK). J Biol Chem.

[24] White CM, Martin K, Lee LF, Haskill JS, Ting JP (1997). Effects of paclitaxel on cytokine synthesis by unprimed human monocytes, T lymphocytes, and breast cancer cells. Cancer Immunol Immunother.

[25] Bogdan C, Ding A (1992). Taxol, a microtubule-stabilizing antineoplastic agent, induces expression of tumor necrosis factor alpha and interleukin-1 in macrophages. J Leukoc Biol.

[26] Janicki-Deverts D, Cohen S, Doyle WJ, Turner RB, Treanor JJ (2007). Infection-induced proinflammatory cytokines are associated with decreases in positive affect, but not increases in negative affect. Brain Behav Immun.

[27] Vollmer-Conna U, Fazou C, Cameron B, Li H, Brennan C, Luck L, Davenport T, Wakefield D, Hickie I, Lloyd A (2004). Production of pro-inflammatory cytokines correlates with the symptoms of acute sickness behaviour in humans. Psychol Med.

[28] Huang Y, Henry CJ, Dantzer R, Johnson RW, Godbout JP (2007). Exaggerated sickness behavior and brain proinflammatory cytokine expression in aged mice in response to intracerebroventricular lipopolysaccharide. Neurobiol Aging.

[29] Harden LM, du Plessis I, Poole S, Laburn HP (2006). Interleukin-6 and leptin mediate lipopolysaccharide-induced fever and sickness behavior. Physiol Behav.

[30] Stracciari A, Guarino M, Crespi C, Pazzaglia P (2009). Hochuekkito improves systemic inflammation and nutritional status in elderly patients with chronic obstructive pulmonary disease. J Am Geriatr Soc.

[31] Hossain MS, Takimoto H, Hamano S, Yoshida H, Ninomiya T, Minamishima Y, Kimura G, Nomoto K (1999). Protective effects of Hochu-ekki-to, aChinese traditional herbal medicine against murine cytomegalovirus infection. Immunopharmacology.

[32] Mori K, Kido T, Daikuhara H, Sakakibara I, Sakata T, Shimizu K, Amagaya S, Sasaki H, Komatsu Y (1999). Effect of Hochu-ekki-to(TJ-41):a Japanese herbal medicine, on the survival of mice infected with influenza virus. Antiviral Res.

[33] Abe S, Tansho S, Ishibashi H, Akagawa G, Komatsu Y, Yamaguchi H (1999). Protection of immunosuppressed mice from lethal Candida infection by oral administration of a kampo medicine, Hochu-ekki-to. Immunopharmacol Immunotoxicol.

[34] Hardin BJ, Campbell KS, Smith JD, Arbogast S, Smith J, Moylan JS, Reid MB (2008). TNF-alpha acts via TNFR1 and muscle-derived oxidants to depress myofibrillar force in murine skeletal muscle. J Appl Physiol.

[35] Reid MB, Lannergren J, Westerblad H (2002). Respiratory and limb muscle weakness induced by tumor necrosis factor-alpha: Involvement of muscle myofilaments. Am J Respir Crit Care Med.

[36] Wajant H, Pfizenmaier K, Scheurich P (2003). Tumor necrosis factor signaling. Cell Death Differ.

[37] Powers SK, Jackson MJ (2008). Exercise-induced oxidative stress: cellular mechanisms and impact on muscle force production. Physiol Rev.

[38] Supinski GS, Callahan LA (2007). Free radical-mediated skeletal muscle dysfunction in inflammatory conditions. J Appl Physiol.

[39] Chen Y, Jungsuwadee P, Vore M, Butterfield DA, St Clair DK (2007). Collateral damage in cancer chemotherapy: oxidative stress in nontargeted tissues. Mol Interv.

[40] Muinelo-Romay L, Alonso-Alconada L, Alonso-Nocelo M, Barbazan J, Aba M (2012). Tumor invasion and oxidative stress: biomarkers and therapeutic strategies. Curr Mol Med.

[41] Akbas SH, Timur M, Ozben T (2005). The effect of quercetin on topotecan cytotoxicity in MCF-7 and MDA-MB 231 human breast cancer cells. J Surg Res.

[42] Mohamed HE, Asker ME, Ali SI, el-Fattah TM (2004). Protection against doxorubicin cardiomyopathy in rats: role of phosphodiesterase inhibitors type 4. J Pharm Pharmacol.

[43] Seyhan N, Canseven AG (2006). In vivo effects of ELF MFs on collagen synthesis, free radical processes, natural antioxidant system, respiratory burst system, immune system activities, and electrolytes in the skin, plasma, spleen, lung, kidney, and brain tissuess. Electromagn Biol Med.

[44] Suzer T, Coskun E, Demir S, Tahta K (2000). Lipid peroxidation and glutathione levels after cortical injection of ferric chloride in rats: effect of trimetazidine and deferoxamine. Res Exp Med (Berl).

[45] Lee MY, Shin IS, Jeon WY, Seo CS, Ha H, Huh JI, Shin HK (2012). Protective effect of Bojungikki-tang, a traditional herbal formula, against alcohol-induced gastric injury in rats. J Ethnopharmacol.

[46] Li X, Wang X, Han C, Wang X, Xing G, Zhou L, Li G, Niu Y (2013). Astragaloside IV suppresses collagen production of activated HSCs via oxidative stress-mediated p38 MAPK pathway. Free Radic Biol Med.

[47] Abe H, Sakaguchi M, Yamada M, Arichi S, Odashima S (1980). Pharmacological actions of saikosaponins isolated from Bupleurum falcatum. 1. Effects of saikosaponins on liver function. Planta Med.

[48] Matsumoto T, Sun XB, Hanawa T, Kodaira H, Ishii K, Yamada H (2002). Effect of the antiulcer polysaccharide fraction from Bupleurum falcatum L. on the healing of gastric ulcer induced by acetic acid in rats. Phytother Res.

[49] Chang WC, Hsu FL (1991). Inhibition of platelet activation and endothelial cell injury by flavan-3-ol and saikosaponin compounds. Prostaglandins Leukot Essent Fatty Acids.

[50] Nose M, Amagaya S, Ogihara Y (1989). Corticosterone secretion-inducing activity of saikosaponin metabolites formed in the alimentary tract. Chem Pharm Bull (Tokyo).

[51] Abe H, Sakaguchi M, Odashima S, Arichi S (1982). Protective effect of saikosaponin-d isolated from Bupleurum falcatum L. on CCl4-induced liver injury in the rat. Naunyn Schmiedebergs Arch Pharmacol.

[52] Hattori T, Ito M, Suzuki Y (1991). Studies on antinephritic effects of plant components in rats (2): Effects of ginsenosides on original-type anti-GBM nephritis in rats and its mechanisms. Nihon Yakurigaku Zasshi.

[53] Grabiec K, Burchert M, Milewska M, Błaszczyk M, Grzelkowska-Kowalczyk K (2013). Systemic and local mechanisms leading to cachexia in cancer. Postepy Hig Med Dosw.

[54] Abaid LN, Lopez KL, Micha JP, Rettenmaier MA, Brown JV, Goldstein BH (2010). Bevacizumab, paclitaxel and carboplatin for advanced ovarian cancer: low risk of gastrointestinal and cardiovascular toxicity. Eur J Gynaecol Oncol.

[55] Kim HJ, Seo JY, Suh HJ, Lim SS, Kim JS (2012). Antioxidant activities of licorice-derived prenylflavonoids. Nutr Res Pract.

[CR56] The pre-publication history for this paper can be accessed here:http://www.biomedcentral.com/1472-6882/14/497/prepub

